# Mechanisms by which Porphyromonas *gingivalis* evades innate immunity

**DOI:** 10.1371/journal.pone.0182164

**Published:** 2017-08-03

**Authors:** Kaveh Abdi, Tsute Chen, Brian A. Klein, Albert K. Tai, Jill Coursen, Xiangdong Liu, Jeff Skinner, Saravanan Periasamy, Youngnim Choi, Benedikt M. Kessler, Robert J. Palmer, Apostolos Gittis, Polly Matzinger, Margaret J. Duncan, Nevil J. Singh

**Affiliations:** 1 Laboratory of Immunogenetics, National Institute of Allergy and Infectious Diseases, NIH, Rockville, Maryland, United States of America; 2 Department of Microbiology, The Forsyth Institute, Cambridge, Massachusetts, United States of America; 3 TUCF Genomics, Tufts University School of Medicine, Boston, Massachusetts, United States of America; 4 Laboratory of Immunoregulation, National Institute of Allergy and Infectious Diseases, NIH, Bethesda, Maryland, United States of America; 5 National Institute of Dental and Craniofacial Research, NIH, Bethesda, Maryland, United States of America; 6 Department of Oromaxillofacial Infection & Immunity, School of Dentistry and Dental Research Institute, Seoul National University, Seoul, Korea; 7 Target Discovery Institute, Nuffield Department of Clinical Medicine Oxford University, Oxford, United Kingdom; 8 Research Technologies Branch, National Institute of Allergy and Infectious Diseases, NIH, Rockville, Maryland, United States of America; 9 University of Maryland School of Medicine, Department of Microbiology & Immunology, Baltimore, Maryland United States of America; New York Medical College, UNITED STATES

## Abstract

The oral cavity is home to unique resident microbial communities whose interactions with host immunity are less frequently studied than those of the intestinal microbiome. We examined the stimulatory capacity and the interactions of two oral bacteria, Porphyromonas *gingivalis* (P. *gingivalis*) and Fusobacterium *nucleatum* (F. *nucleatum*), on Dendritic Cell (DC) activation, comparing them to the effects of the well-studied intestinal microbe Escherichia *coli (E*. *coli)*. Unlike F. *nucleatum* and E. *coli*, P. *gingivalis* failed to activate DCs, and in fact silenced DC responses induced by F. *nucleatum* or E. *coli*. We identified a variant strain of P. *gingivalis* (W50) that lacked this immunomodulatory activity. Using biochemical approaches and whole genome sequencing to compare the two substrains, we found a point mutation in the *hagA* gene. This protein is though to be involved in the alteration of the PorSS/gingipain pathway, which regulates protein secretion into the extracellular environment. A proteomic comparison of the secreted products of the two substrains revealed enzymatic differences corresponding to this phenotype. We found that P. *gingivalis* secretes gingipain(s) that inactivate several key proinflammatory mediators made by DCs and/or T cells, but spare Interleukin-1 (IL-1) and GM-CSF, which can cause capillary leaks that serve as a source of the heme that P. *gingivalis* requires for its survival, and GM-CSF, which can cause epithelial-cell growth. Taken together, our results suggest that P. *gingivalis* has evolved potent mechanisms to modulate its virulence factors and dampen the innate immune response by selectively inactivating most proinflammatory cytokines.

## Introduction

Humans harbor diverse microbial communities in various tissues, and these microorganisms can dramatically alter the local and systemic immune functions. The bi-directional interactions between the microbiome and the immune system have been extensively studied in the gut and skin, but relatively little is known about such events in the oral cavity [1–3], which is exposed to an extraordinarily wide variety of external agents. In addition to encounters with food, air and fluids from other individuals, the microbiome of the oral cavity includes over 700 species of microbes, some of which are thought to be involved in pathological conditions ranging from caries to cancer [4–7]. Among the oral microbiota, Porphyromonas *gingivalis* (P. *gingivalis*) is relatively well studied because of its association with chronic inflammation of gum tissue (periodontitis) [8, 9]. Although P. *gingivalis*, by itself is not sufficient to cause gingivitis or bone loss [10], it has been classified as “keystone pathogen” [11] based on the findings that it seems to be the essential component of the microbial community that causes oral disease [10].

An intriguing possibility is that P. *gingivalis* sets the stage for the entire cascades of disease by altering the local immune microenvironment, thus allowing pathobionts to subsequently colonize or overgrow [12]. There are supportive evidence for this notion that has come from studies on P. *gingivalis* interactions with cells of the immune system. It had been suggested that P. *gingivalis* Lipopolysaccharide (LPS) skews the immune responses away from an inflammatory T_H_1-type response and towards a semi-T_H_2-like response [13, 14]. There are also data showing that P. *gingivalis* can directly act on complement system through its arginine-specific gingipains to block antimicrobial response [10, 15–17]. LPS is not the only molecule in a bacterium’s arsenal. We therefore asked if intact P. *gingivalis* might have even stronger effects on the activation of Dendritic Cells (DCs), one of the earliest immune cells to be activated in an immune response.

Because P. *gingivalis* has been shown to need companion or “bridge” organisms [10], we also used the oral commensal Gram-negative anaerobe, F. *nucleatum* that co-aggregates with, and promotes the colonization of periodontitis-associated bacteria such as P. *gingivalis* [18, 19]. Although F. *nucleatum* primarily colonizes the human oral cavity, it has been associated with diseases of various other organs, including periodontal, lung, abdominal and gynecological infections [20–23]. We postulated that comparing the two oral microbes for their direct impact on DCs, alone and in combination, might offer some insight into how individual components of the oral microbiota might contribute to control DC activation and its downstream consequences on T cell effector functions.

Using phenotypic and functional assays, we showed that P. *gingivalis* induced very low levels of inflammatory gene expression in DCs, in contrast to F. *nucleatum* and E. *coli*, which were potent activators. In addition, P. *gingivalis* inactivated a broad assortment of human and mouse cytokines and chemokines that are normally produced by activated DCs, thus further dampening the inflammatory cascade. We compared a mutant strain of P. *gingivalis* (W50) to the standard strain obtained from American Type Culture Collection (W50-ATCC). We discovered that a mutant strain has markedly stronger immunosuppressive properties compared to W50-ATCC, and was able to degrade a wide variety of mouse and human cytokines and chemokines.

Taken together, our data suggest that P. *gingivalis* has evolved the ability to operate somewhat under the immunological radar. It is a very weak activator of DCs. It also dampens DC activation by other bacteria, and it backs that up with the ability to degrade almost all of the cytokines and chemokines released by activated DCs and T cells. Such immunomodulatory mechanisms may underlie P. *gingivalis* ability to operate as a “keystone pathogen” in the oral cavity.

## Materials and methods

The authors have declared no conflicts of interest.

### Mice

Adult 8-14-wk-old male and female B10.A RAG2^-/-^, mice and 5C.C7 RAG2^-/-^ TCR transgenic mice [containing T cells specific for peptide 88–103 of moth cytochrome *c* (MCC)] were purchased from the NIAID/Taconic Farms, Inc. exchange. Mice were housed in the NIH animal facilities (an AAALAC accredited facility) in a specific pathogen-free barrier colony that is certified by the United States Office of Laboratory Animal Welfare (OLAW) under the guidance of the Public Health Service (PHS) Policy on Humane Care and Use of Laboratory Animals. All studies were carried out in accordance with protocols LIG-32 and LCMI-14E approved (5/01/2013) by the Institutional Animal Care and Use Committee (IACUC) of the NIAID, NIH. All experimental protocols involving mice in this paper were specifically stated and approved by the IACUC in LIG-32 or LCMI-14E. The mice euthanasia protocol approved under this protocol involves CO2 asphyxiation followed by cervical dislocation. In general mice are placed in a 20% Co2 displacement unit until they are unconscious and then the Co2 is increased to 100%, followed by cervical dislocation as a secondary method.

### Media and reagents

Bacterial Lipopolysaccharide (LPS) was derived from E. *coli* Serotype 0127:B8 (Sigma Aldrich). All recombinant cytokines were obtained from Peprotech Inc. Cells were cultured in complete medium [(Iscove’s Modified Dulbecco’s Medium (IMDM: GibcoBRL)] supplemented with 10% heat-inactivated Fetal Bovine Serum (FBS: tested to be free of endotoxin, mycoplasma, virus, and bacteriophage; GibcoBRL), L-glutamine, 55 μM 2ß-Mercaptoethanol (GibcoBRL), penicillin, streptomycin, and gentamicin (Biosource). The antibiotics prevented any bacterial growth in our cultures, even when we added large numbers of living bacteria.

### Cytokine measurements

Cytokines in the culture supernatant (CSN) were quantitated using SearchLight Multiplex cytokine array (Aushon Biosystems, Billerica, MA). IL-12 heterodimer (IL-12p75) was specifically detected by using the ELISA kit Quantikine (R&D Systems).

### Generation of BMDCs

Briefly, bone marrow cells were flushed out of the femurs and tibias of 8-12-wk-old mice into complete medium and pipetted vigorously to make a single cell suspension, then passed through a cell strainer (70-μm Nylon mesh; BD Falcon ^TM^). Erythrocytes were lysed using ACK lysis buffer (Biosource) and cells washed 2x with complete medium and cultured at 1x10^6^ cells in 2ml/well in a 24-well plate with complete medium supplemented with 30U/ml GM-CSF and 60U/ml IL-4 for 6 days as described previously [24].

### *In vitro* generation of antigen-activated/primed 5C.C7 CD4^+^ T cells

Spleens from B10.A/SgSnAi TCR–Cyt 5C.C7-1 RAG-2^-/-^ mice were dissociated into homogenous single cell suspensions and passed through a cell strainer (70-μm Nylon mesh) from BD Falcon^TM^. Erythrocytes were lysed using ACK lysis buffer (Biosource). Splenocytes were washed 3x in cold complete medium and adjusted to 1x10^6^/ml, then cultured in complete medium plus 1 μM of the Moth Cytochrome *C* (MCC) 88–103 peptide at 37°C with 5% CO_2_. Fresh medium was added after 3 days of culture. After 5 days, the cultured cells were harvested, washed 2x with medium, and re-cultured in the absence of peptide with 10–20 U/ml of recombinant mouse (rm) IL-2. The medium was replenished every 5–7 days with fresh medium plus rmIL-2. For T-DC coculture experiments, these CD4^+^ T cells were purified with a CD4^+^ T cell isolation kit containing anti-CD8α, CD11b, CD45, DX5, and Ter-119 mAbs for depletion (Miltenyi Biotech.) with the addition of anti-CD11c and anti-class-II (Miltenyi Biotech). We used 3–4 LS columns in tandem instead of using LD columns to deplete the cells. These T cells, which were >95% CD4^+^, were used at various days of culture as a source of *in vitro* primed/antigen-activated T cells.

### DC/T cell coculture

1x10^5^ bead depleted (CD4^+^ T cell isolation kit, Miltenyi Biotech) cultured 5C.C7 CD4^+^ T cells were incubated with 2x10^4^ BMDCs/well from B10.A RAG^-/-^ mice ±0.1μM moth cytochrome *c* (MCC) peptide 88–103 in triplicate in 96-well u-bottom plates in a final volume of 200 μl/well for 48 h. The supernatants were removed and kept frozen at –30°C before measuring the cytokines by ELISA.

### DC/bacteria coculture and treatment

In coculture experiments, 5x10^7^ bacteria were added to the 24-well plate containing BMDCs for 16 h. For bacterial supernatant (SN) preparation, ~10^11^ bacteria were harvested in the exponential stage of growth, washed twice with PBS, and incubated at 37°C in 5 ml PBS for 1 h. The cells were then spun at 4500 rpm for 15 min and the SN filtered through a 0.22-μm syringe filter before being used (bacteria SN). In order to treat cytokines, 200 μl of the bacteria SN or ~10^9^ whole bacteria were added to 1 ml of complete medium (containing 10% FCS) spiked with various cytokines and kept at 37°C for 24 h before quantitating residual cytokines with cytokine array.

### FACS analysis

All steps were done on ice or in 4°C centrifuges to prevent the sudden upregulation of MHC class II caused by handling [25]. Plates containing DC cultures were cooled for 20 min. The cells were then gently harvested, spun, counted and distributed into 96 well plates at about ~10^6^ per well. We added 10 μl “ultrablock” (a mixture of 1:1:1 mouse, rat and goat serum containing 10μg/ml of the monoclonal anti-FcR antibody 2.4G2) for 10 min before adding a master-mix of a combination of antibodies anti-mouse CD80, CD40, CD11c and MHC class II (BD Pharmingen). Each antibody was titrated for optimal signal-to-background before use. After 20 min, the cells were washed and resuspended in PBS containing the viability dye, 7AAD plus 1% FCS, for 5 min, then centrifuged and resuspended in FACS buffer for analysis.

### Bacteria culture and strains

P. *gingivalis* used in this study 53978 (W50), BAA-308 (W83), ATCC33277, and F. *nucleatum*, were obtained from American Type Culture Collection (ATCC). P. *gingivalis* W50-NIDCR was a generous gift from Dr. P. Kolenbrander in the National Institute of Dental and Craniofacial Research (NIDCR). All oral bacteria were grown anaerobically (GasPak EZ; BD, Sparks, MD) in Bacto^TM^ Brain Heart Infusion (BD, Sparks, MD or Anaerobe Systems Morgan Hill, CA), supplemented with 5 mg/L hemin and 1 mg/L vitamin K. E. *coli* were grown in *Luria Bertani Broth* (K.D Medical Columbia, MD). Bacteria were harvested in the exponential stage of their growth and washed 2x with PBS before being used at an O.D. of 600 nm.

### Heat-killed *P*. *gingivalis*

*P*. *gingivalis* was harvested in the exponential stage of growth and immediately incubated in a 65°C water bath for 1 h and washed 2x with PBS before coculturing with BMDCs.

### Gingipain enzymatic assay (Rgp)

To assess the gingipain activity of P. *gingivalis* strains, 20 μl of bacterial supernatant was mixed with Nα-Benzoyl-DL-arginine β-naphthylamide hydrochloride (BANA) substrate and 100 μl of 100 mM Tris-HCl buffer pH 7.4 (with 2mM dithiothreitol), and incubated at 37°C for 2 h. Optical densities at 410 and 600 nm were measured every 5 min. using a PowerWave HT (Biotek) spectrophotometer. Assays were performed three times in triplicate.

### Two-dimensional difference in gel-electrophoresis and one-dimensional gel

2.5 ml of cell-free bacterial SN were concentrated (10x) using Amicon Ultra-4 (10,000 MWCO) (Millipore) by spinning at 2500xg for 15 min at 4°C. The concentrated SN was separated by SDS-PAGE for one-dimensional gel and visualized by Coomassie or silver staining and two-dimensional gel was analyzed by a service contract (Applied Biomics, Hayward CA) as previously described [26]. Given the robustness of the biological/enzymatic assays we observed, the proteomic analyses were performed once each.

### Quantitative RT-PCR

BMDCs were cultured in a 24-well plate for 6 h with 10^8^ bacteria/well and RNA was isolated from the cells by RNeasy kit (Qiagen). RNA was treated to remove DNA contamination (Invitrogen) and the cDNA was synthesized using random primers, and reverse transcribed using Superscript III (Invitrogen). Real-time PCR analyses were performed for expression of IL-1 α, IL-1β, TNF α, IL-6, IL-12p40, IL-10, IL-23p19, IL-12p35, and using Taqman gene expression primers and probes mix (Applied Biosystems). The mRNA expression levels were calculated as dCt = Target-Actin B/nono, where Actin B/nono was the endogenous control.

### Mass spectrometry and data analysis

Peptides were identified from mass spectroscopy (MS) data using SEQUEST algorithms and a minimum of two peptides. Peptide thresholds of 95% were used for identification as previously described [26].

### Statistical analysis

All statistical analyses were performed using Prism (GraphPad Software).

## Results

### *P*. *gingivalis* is a poor activator of DCs

**Fig 1:**
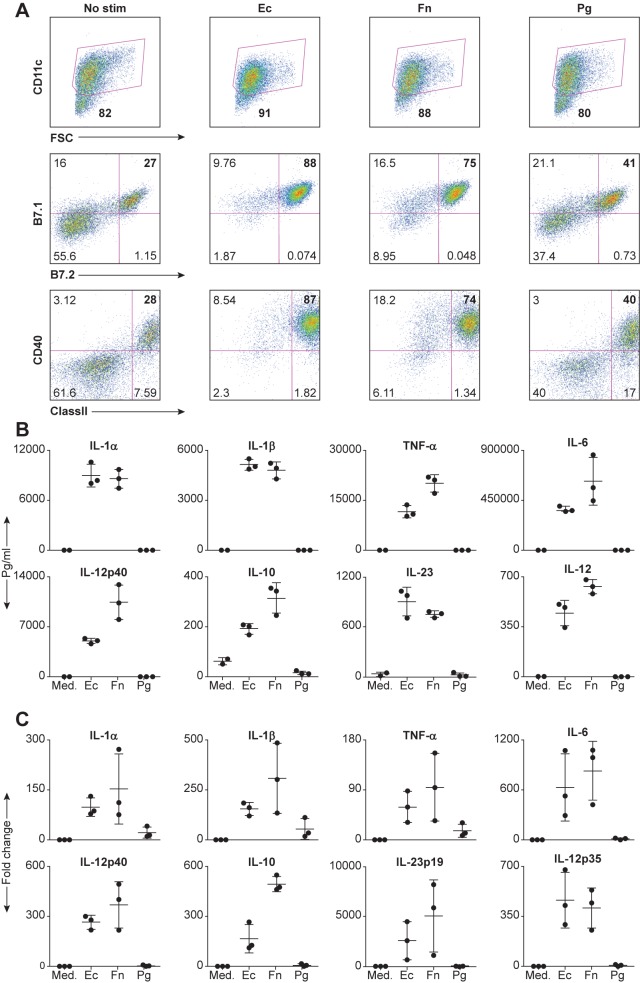
Inflammatory cytokine production from DCs is extinguished in the presence of *P*. *gingivalis*. (**A**) DCs from B10.A-Rag2^-/-^ mice were either unstimulated (medium) or stimulated on the sixth day with 5x10^7^ bacteria in a 24-well plate for 16h at 37°C. Cells were harvested, stained with directly labeled monoclonal antibodies and analyzed by FACS analysis. Cells were gated for the CD11c positive population and analyzed for the costimulatory molecules B7.1, B7.2 and CD40, and the antigen-presenting molecule, MHC class II. Numbers indicate the percentage of the population in the gate or quadrant (**B**) Same as (A) Culture CSN were analyzed for the presence of various cytokines using Searchlight protein arrays. (**C**) Same as (B) except the co-culture period was 6h, at which time we isolated total RNA using the RNeasy kit and analyzed it by Real-time RT-PCR. The data are expressed as the mean ± SD of three independent experiments.

It is well established that DCs respond to various bacterial products from gut bacteria such as E. *coli* by upregulating cell surface costimulatory molecules and rapidly secreting cytokines and chemokines. To compare the ability of oral bacteria to elicit these functional responses by DCs, we used bone marrow-derived DCs (DCs) obtained from Recombinant Activating Gene-2 deficient mice (RAG2^-/-^), which lack T and B cells, to eliminate the possibility of B and/or T cell contamination (which might alter the function of the DCs). We stimulated the DCs with P. *gingivalis* and F. *nucleatum*, two different oral bacteria species that have been shown to co-aggregate *in vitro* [18, 27], and with E. *coli* and assessed their activated status 16 hours later. Fig 1A shows that nearly 100% of the DCs stimulated with E. *coli* upregulated the co-stimulatory molecule B7.1, and nearly 90% upregulated B7.2, CD40 and the antigen-presenting molecule, MHC class II. The oral anaerobe F. *nucleatum* was slightly less active, while P. *gingivalis* stimulated only about 12–15% of the DCs over the background.

We saw dramatic differences when we analyzed the secretion of cytokines by the activated DCs. Fig 1B shows that P. *gingivalis* failed to induce any of these cytokines from DCs, while both E. *coli* and F. *nucleatum* were potent inducers. In some cases, the cytokine levels in P. *gingivalis*-stimulated cultures were even lower than those secreted by the untreated controls.

To begin to uncover the mechanism by which P. *gingivalis* blocks cytokines, we started by determining whether it can block transcription. We used qRT-PCR to analyze the amount of cytokine mRNA in DCs after 6 hrs of stimulation with various bacteria. As shown in Fig 1C, both E. *coli* and *F*. *nucleatum* induced greater than 100-fold changes in the mRNA expression of various cytokines in DCs. In contrast, P. *gingivalis* induced only very low levels of mRNA for IL-1 α, IL-1 β and TNF α, and no mRNA for the other cytokines. Thus, P. *gingivalis* is a very poor activator of DCs.

**Fig 2:**
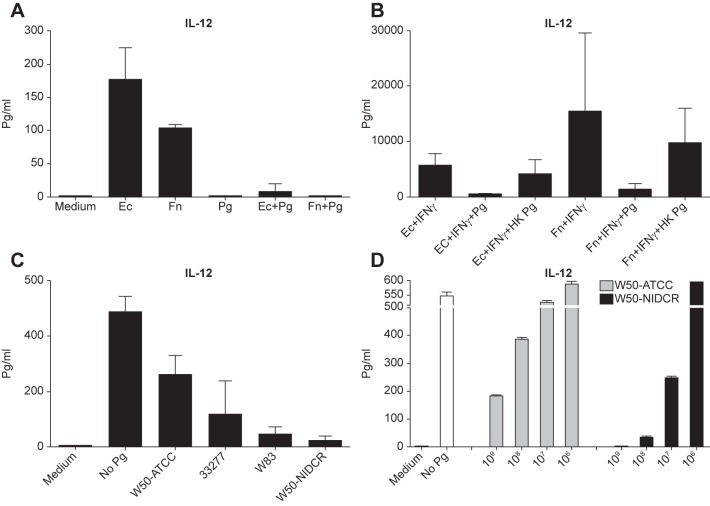
Differential effect of *P*. *gingivalis* W50 substrains on extinguishing cytokine production from DCs. **(A)** DCs from B10.A-Rag2^-/-^ mice were either unstimulated (medium) or stimulated on the sixth day with 5x10^7^ E. *coli*, F. *nucleatum* or P. *gingivalis* alone or a mixture of two bacteria together in a 24-well plate for 16 h at 37°C. CSN were analyzed for the presence of IL-12p75 using a specific ELISA. **(B)** Same as (A) except that IFNγ was added to the cultures to enhance IL-12 production. P. *gingivalis* were heat-killed at 60°C for 1 h in a water bath (HKP*g)*, or not, before being added at 5x10^7^/well to the culture containing F. *nucleatum* or E. *coli*. **(C)** DCs were stimulated with 100 ng/ml of LPS (*E*. *coli*) plus 100 ng/ml of IFNγ alone (No *Pg*) or in combination with various strains of P.*gingivalis* at 5x10^7^/well for 24 h before testing for the presence of IL-12p75 in the CSN. **(D)** Same as (C) except various numbers of P. *gingivalis* W50-NIDCR or -ATCC were added to the DCs in a 24-well plate. (A-D) Data are the mean ± SD of compilation of two independent experiments. (D) The data are expressed as the mean ± SD of duplicates.

To determine whether P. *gingivalis* was merely a weak activator of DCs or whether it might actually be suppressive, we asked whether P. *gingivalis* could modulate cytokine secretion by DCs activated by E. *coli* or F. *nucleatum*. We stimulated DCs with E. *coli* or F. *nucleatum*, plus IFNγ(strong stimulus for IL-12 production) [28], either alone or in combination with 5x10^7^ P. *gingivalis* bacteria and found that the addition of P. *gingivalis* severely impaired the levels of IL-12 produced by the DCs (Fig 2A). The bacterium did not simply kill the DCs, as they remained viable (excluded Trypan blue) by stimulating T helper cells and receive help (S1 Fig). In addition, because it had been shown previously that the LPS of P. *gingivalis* does not stimulate strong inflammatory responses from DCs [13], we asked if the suppressive activity of P. *gingivalis* was due to its unique LPS [29] by asking if it was sensitive to heat. Fig 2B shows that the inhibitory activity was markedly reduced in DC cultures given heat-killed P. *gingivalis*. Thus, in addition to having a unique LPS, P. *gingivalis* also expresses a suppressive activity that is heat-labile, suggesting that the suppressive activity is protein.

### Immune modulation by P. *gingivalis* shows strain-dependent phenotypic polymorphisms

To identify the genetic loci associated with P. *gingivalis* suppressive function, we examined several strains of P. *gingivalis* to determine whether there might be genetic variation among them that would allow us to locate relevant gene(s). Thus far, we had used a W50 strain [a gift of Dr. P. Kolenbrander, National Institute of Dental and Craniofacial Research, (NIDCR)], referred to hereafter as W50-NIDCR. To test whether the suppressive activity was unique to this strain, we compared it to three other strains of P. *gingivalis* (W83, W50, and 33277) from the American Type Culture Collection (ATCC). We stimulated DCs overnight with E. *coli* LPS plus IFNγ [28], in the absence or presence of each P. *gingivalis* strain, and measured IL-12 protein in the CSN.

Fig 2C shows that W50 from ATCC (hereafter refer to as W50-ATCC) was considerably less potent than W50-NIDCR at reducing IL-12 production (Fig 2C), while strain W83 was only slightly less inhibitory than W50-NIDCR. Fig 2D shows that, when the two different W50 *P*. *gingivalis* were titrated, W50-NIDCR was almost 100 times more potent than W50-ATCC at inhibiting IL-12 production.

### Whole genomic sequencing and comparison of P. *gingivalis* W50-NIDCR and W50-ATCC

The evolutionary adaptation of a microbe to its host often involves multiple genetic modifications. To determine the mechanism by which W50-NIDCR was able to potently modify its virulence factors, we took a genomic approach to compare the four *P*. *gingivalis* strains and as a control, we compared them to *E coli*.

**Fig 3:**
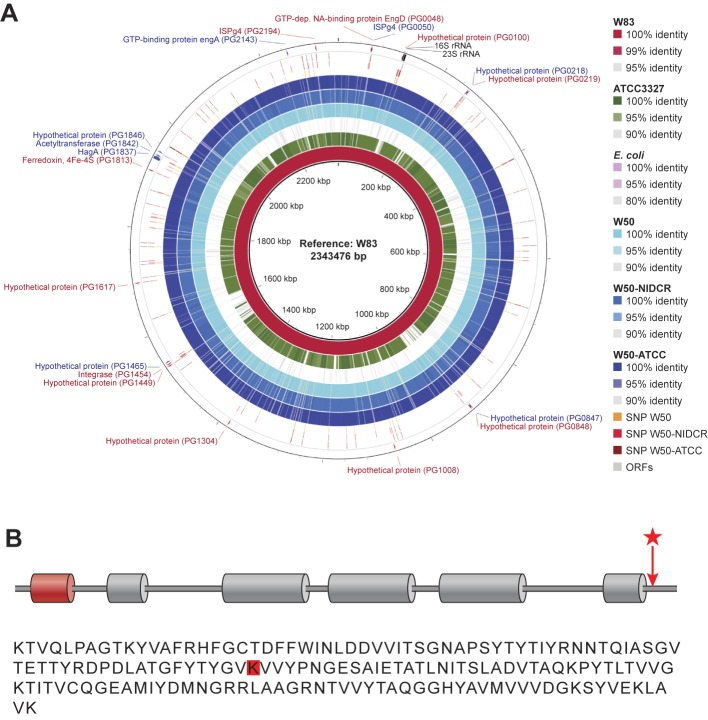
Mutation in W50-NIDCR HagA causing alteration in the PorSS/gingipain pathway. (**A**) Draft genomes of NCBI W50 and two W50 variant strains (W50-ATCC and W50-NIDCR) were mapped to the reference genome of W83 to illustrate the sequence similarities between these two genomes and the reference. As controls, genomes of *P*. *gingivalis* 33277 and *E*. *coli* were also included in these analyses. The three outer rings (SNP W50, SNP W50-NIDCR, and SNP W50-ATCC) show the SNP loci indentified by the software *“breseq*.*”* Those that are unique among the three genomes are shown with the +/- affected genes and their annotations in the outer most ring (ORFs) and their annotations. The map was drawn using “BRIG” genome alignment software. (**B**) Red cylindrical domain is a peptidase C25 C-terminal domain, grey cylindrical domains are cleaved adhesin domains, and the red star/arrow indicates the location of W50-NIDCR SNP. Not depicted are the Fibronectin Type III domains, which encompass the cleaved adhesin but extend further in the N- and C-terminal directions. K75* in EIW91729.1 (partial PrtH domain) is analogous to K2074* in AAQ66831.1 (HagA). SNP occurs within the Fibronectin Type III domain at N-terminus of the protein. The raw sequences used in this study have been deposited to the NCBI Short Reads Archive (SRA) under the umbrella BioProject ID PRJNA302472 (*http://www.ncbi.nlm.nih.gov/bioproject/PRJNA302472*).

First, we sequenced the whole genomes of W50-NIDCR, W50-ATCC and ATCC 33277, and assembled contigs using the reference genome from the previously sequenced W83 strain (Fig 3A). Across the entire genome of the W50-NIDCR, we were able to identify unique single nucleotide polymorphisms (SNPs) in 20 different loci by BRESEQ analysis. Two of these loci were the 16S and 23S rRNA, while SNPs in 4 of the other loci fell into non-coding or intergenic regions. Thirteen of the remaining 14 loci included a high concentration of SNPs in multiple predicted protein coding regions with yet unknown function.

The 14^th^ was a SNP difference in the coding region of the *hagA* gene, which encodes hemagglutinin A, a 280-kDa protein encoded by an 8 Kb gene containing multiple large direct repeat units [30, 31]. A single A→T transversion in this locus in W50-NIDCR resulted in a codon change (AAA to TAA) yielding a premature stop codon after amino acid 75 in the open reading frame of the *HagA* gene (Fig 3B).

### The differential proteome of strains W50-NIDCR and W50-ATCC reveal alterations in the proteolytic machinery

HagA is an outer membrane protein that has cysteine proteinase adhesin domains involved in adherence to host cells and to other bacteria [32, 33]. It is also a major virulence determinant in P. *gingivalis* [34]. Moreover, during periodontal disease, antibodies targeted the protein products of HagA [35–37]. In addition to its role in adhesion, there is some evidence that HagA may have a regulatory effect on the processing and/or secretion of proteins [38]. We therefore used a proteomic approach to look for proteins that might be differentially secreted by W50-NIDCR and W50-ATCC.

**Fig 4:**
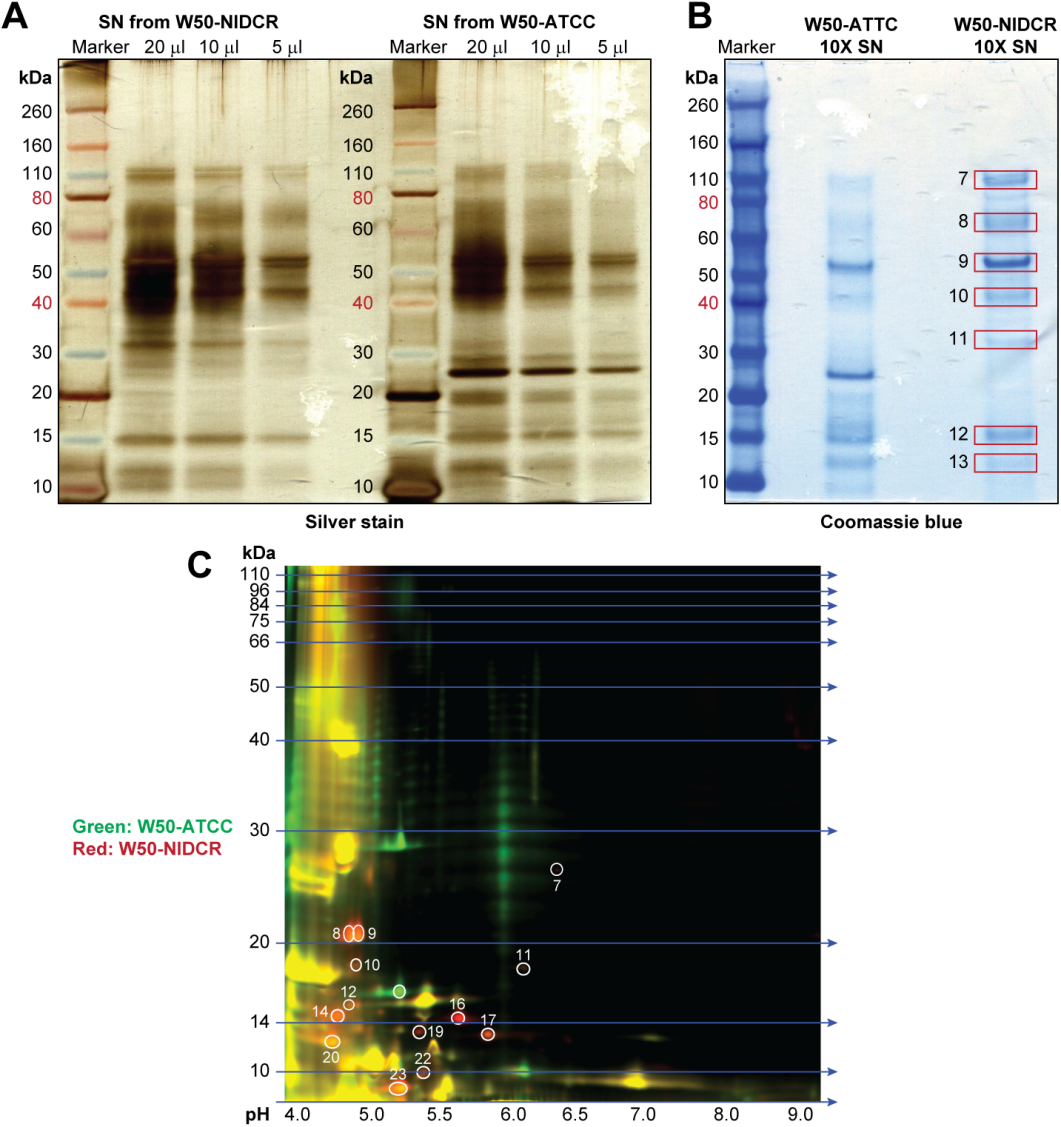
Comparative proteomic of particle-free SN from W50-NIDCR and W50-ATCC. (**A**) Various amounts of particle-free SN from W50-ATCC and W50-NIDCR were resolved by one-dimensional SDS-PAGE and protein bands were visualized by Silver staining. (**B**) Coomassie staining of the same SNs that had been concentrated to 10X as described indicated in the materials and methods. (**C**) Same as (A) except particle-free bacterial SN from W50-ATCC and W50-NIDCR were labeled green or red, respectively, and resolved by two-dimensional difference in-gel electrophoresis.

We compared cell-free SNs from each strain, resolved the proteins on one-dimensional SDS-PAGE and revealed by silver staining (Fig 4A). This preliminary analysis suggested that there were indeed unique differences in proteins of different molecular weights secreted by each strain. To further identify these proteins, we concentrated the SNs and visualized the resolved protein bands by Coomassie blue staining (Fig 4B). We excised total of 13 spots (Coomassie gel) from W50-ATCC and W50-NIDCR combined and processed them for identification by liquid chromatography-tandem mass spectrometry (LC-MS). However, we focused only on those proteins that unique to W50-NIDCR in comparison to W50-ATCC, which are marked 7–13 (Fig 4B) while the complete comparison is listed in S2 Fig. These bands contained peptides from both arginine- and lysine-specific gingipains (Table 1), a complex set of “trypsin-like” cysteine and arginine proteases that contribute to the degradation of various host proteins [39, 40]. One of these proteins (immunoreactive 61 kDa antigen PG91) was found in multiple bands (bands 7–13) with no known function. The reason for the presence of this protein in multiple bands is indicative of either posttranslational modification of PG91 or that it might be a target of other active proteolytic enzymes in W50-NIDCR (Table 1). The other non-gingipain protein identified in the W50-NIDCR supernatant was Omp28—an outer membrane protein expressed by a wide variety of Gram-negative bacteria, including P. *gingivalis* isolates [41]. Many outer membrane proteins are part of the secretory system (small size vesicles 20–500 nm), which are the means by which bacteria disseminate their virulence factors into the extracellular milieu to modulate host immune responses [42], and have been shown to contain gingipain and other proteolytic enzymes [43].

**Table 1:** Summary of proteins identified using Mass Spectrometry of 1D gel bands comparing W50-NIDCR and W50-ATCC. Ranks 3–9 are unique to W50-NIDCR.

Rank	Accession	Protein	Spots	Total number of Peptides
1	CAA10226.1	RagA	7,8,9,10,11	135
2	Q51817.1	Lys-gingipain	7,8,9,10,11,12,13	270
3	AAD51076.1	Immunoreactive 61kD antigen PG91	7,8,9,10,11,12,13	102
4	AAD51843.1	Outer membrane protein Omp28A	11	18
5	AAD51078.1	Immunoreactive 84kD antigen PG93	8	8
6	P0AA25.2	Thioredoxin (Trx1)	8	4
7	AAF03904.1	Heme-binding protein FetB	11	2
8	Q7MXT8.1	Bifunctional enzyme LpxC/FabZ	12	2
9	AAG24228.1	Putative outer membrane protein PG57	13	2

We decided to further characterize the proteome of the SN obtained from the two W50 strains at higher resolution using two-dimensional difference in gel-electrophoresis (2D DIGE). Therefore, we analyzed the two W50 SNs by labeling them green for W50-ATCC and red for W50-NIDCR (Fig 4C). The protein spots that showed differential abundance between W50-ATCC and W50-NIDCR were excised and subjected to LC-MS analysis. Table 2 lists a number of distinct proteins (13 spots containing at least 3 peptides. comprising 35 proteins) that were highly enriched in W50-NIDCR (orange and red spots) compared to W50-ATCC (green). Like the 1D analysis, the 2D gel data showed that the supernatant of W50-NIDCR showed more gingipain when compared to the supernatant of W50-ATCC, and that many of the 35 proteins identified from the SN were either gingipain or involved in the processing of gingipain (Table 2). This analysis also revealed an over-expression of several proteases that are associated with the secretory system (PorSS/gingipain pathway). These include multiple gingipains (Rgp and Kgp), PrtH domain proteases, various hemagglutinins, and the majority of these proteins contained C-terminal domains (CTDs) that are usually processed by PorSS/gingipain pathways.

**Table 2:** Summary of proteins identified by tandem mass spectrometry from 2D gel. Differential bands were excised from W50-NIDCR (highly enriched red/orange spots) and identified using mass spectrometry (MS). The spots corresponding to each protein, and the total number of peptides identified as belonging to the protein during the MS analyses, are shown in columns 3 and 4.

Accession	Protein	Spots	Total number of Peptides
WP_005874718.1	SusC/RagA family TonB-linked outer membrane protein	9,14,20,23	37
AAB49691.1	hemagglutinin	22 & 10	17
P46071.1	Protease PrtH	10 & 22	17
AAX47719.1	RagA3	8,9,20	14
WP_012457436.1	T9SS C-terminal target domain-containing protein	7	13
XP_001386199.1	hypothetical protein PICST_73499	19	13
WP_005873522.1	zinc carboxypeptidase	7	12
WP_004583425.1	membrane protein	12 & 14	11
WP_012457815.1	collagen-binding protein	19	11
AAX47723.1	RagA2	9, 11, 20	11
WP_012457400.1	SusC/RagA family TonB-linked outer membrane protein	9,10,20	11
AAA69539.1	Arg-gingipain-1 proteinase	22	10
AAC18876.1	arginine-specific thiol protease precursor	22	9
WP_010956404.1	hypothetical protein	7	9
XP_001010368.1	hypothetical protein TTHERM_00354700	20	9
WP_012457845.1	hypothetical protein (old name TPR domain protein)	16	9
WP_012458653.1	peptidase C25 (old name arginine-specific cysteine proteinase RgpA)	22	8
AAD01810.1	hemagglutinin/protease	22	8
WP_004585461.1	hypothetical protein	16	8
WP_010956068.1	peptidase (old name extracellular protease)	12	7
P72197.1	Lys-gingipain, catalytic subunit;39 kDa adhesin	10	7
Q51817.1	Lys-gingipain W83; PrtK48; Lys-gingipain catalytic subunit	10	7
CAA57997.1	protease precursor	22	7
AAX47715.1	RagA4	12 and 9	7
WP_012458254.1	peptidase	12	6
YP_003987329.1	putative ankyrin repeat protein	23	6
ZP_04009894.1	cell wall surface anchor family	16	5
CAN81791.1	hypothetical protein VITISV_020569	20	5
Q51817.1	Lys-gingipain W83; Lys-gingipain catalytic subunit;39 kDa adhesin	22	5
CAA68897.1	TonB-linked adhesin	22	5
AAR37428.1	hypothetical protein MBMO_EBAC750	20	4
AAS68176.1	Lys-gingipain	22	4
NP_721075.1	methyltransferase	23	4
WP_015836135.1	hypothetical protein	20	3
WP_005874011.1	hypothetical protein	17	3

### Supernatant from P. *gingivalis* W50-NIDCR but not W50-ATCC contains high levels of arg-gingipain activities

**Fig 5:**
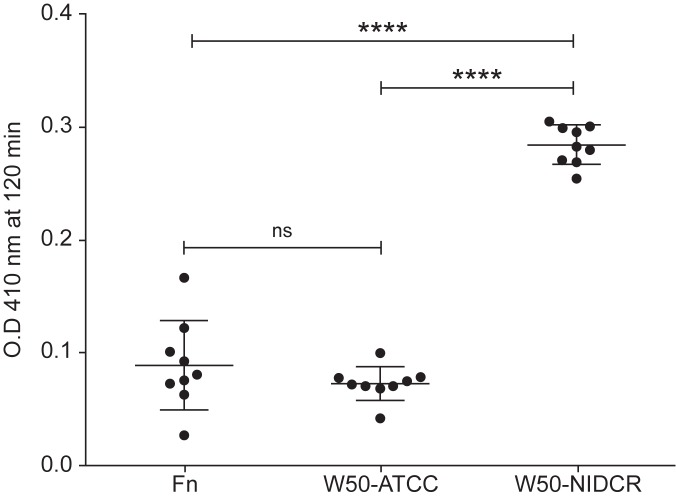
Measuring arginine gingipain in the bacterial SN using a gingipain-specific enzyme assay. 20 μl of supernatant from various bacteria were mixed with the substrate Nα-Benzoyl-DL-arginine β-naphthylamide hydrochloride substrate in 100 μl of Tris buffer plus dithiothreitol (D.T.T) and incubated at 37° C for 2 h. Optical density at 410 and 600 nm were measured with a spectrophotometer. These data are expressed as the mean ± SD of multiple wells and are representative of two independent experiments. Significance was tested using a one-way ANOVA. ****P< 0.0001

To directly test the levels of gingipain in the supernatants of W50-NIDCR, W50-ATCC, (and F. *nucleatum* as a control) we used an enzyme assay specific for Arg-gingipains (Rgp) [44] and found that the SN from W50-NIDCR contained significantly more gingipain than the supernatants of W50-ATCC and F. *nucleatum* (Fig 5). Importantly, various P. *gingivalis* strains possess membrane-associated gingipain activities on the cell surface, and secretion of gingipain from P. *gingivalis* has not been widely reported yet [40] except in one strain (HG66) [45, 46]. Collectively, these data suggest that the mutation in W50-NIDCR results in an alteration in PorSS/gingipain secretory pathway and increased secretion or translocation of gingipain from the bacterial outer membrane and its accumulation in the extracellular environment. Alternatively, it is also plausible that this mutation affects gingipain surface attachment. These would need future studies to clarify

### Supernatants from P. *gingivalis* cultures degrades mouse and human cytokines and chemokines

**Fig 6:**
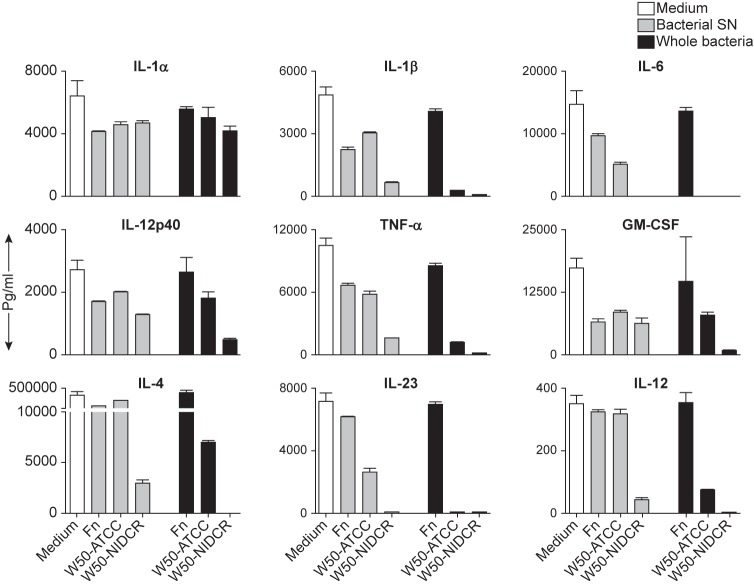
Supernatants of P. *gingivalis* destroy mouse cytokines. Various recombinant mouse cytokines were pooled and then spiked into a single sample of 800 μl of complete medium (contained 10%FCS) in a 24-well plate in the absence (medium) or presence of 200 μl of particle-free bacteria SN or ~1x10^8^ whole bacteria and incubated at 37°C for 22 h, at which time the CSN was tested for the presence of cytokines using a multiplex cytokine array. These data are expressed as the mean ± SD of duplicates and are representative of two independent experiments.

The data so far suggested that the lack of cytokine production we had seen at the mRNA level might not be the only mechanism by which P. *gingivalis* suppresses immunity, but that P. *gingivalis* seems to actively secrete proteases that act directly on the cytokines produced by activated immune cells. To test this, we obtained a large set of recombinant mouse cytokines, diluted them to known concentration, pooled them and spiked the sample with either cell-free culture supernatants or intact bacterium from F. *nucleatum*, W50-ATCC and W50-NIDCR, incubated them for 22 hours at 37°C, and measured the remaining cytokine levels. Fig 6 show that the supernatants from W50-NIDCR destroyed all of the tested cytokines except IL-12p40, GM-CSF and IL-1 α. The intact bacteria were even more potent, destroying both IL-12p40 and GM-CSF while still having little effect on IL-1 α.

**Fig 7:**
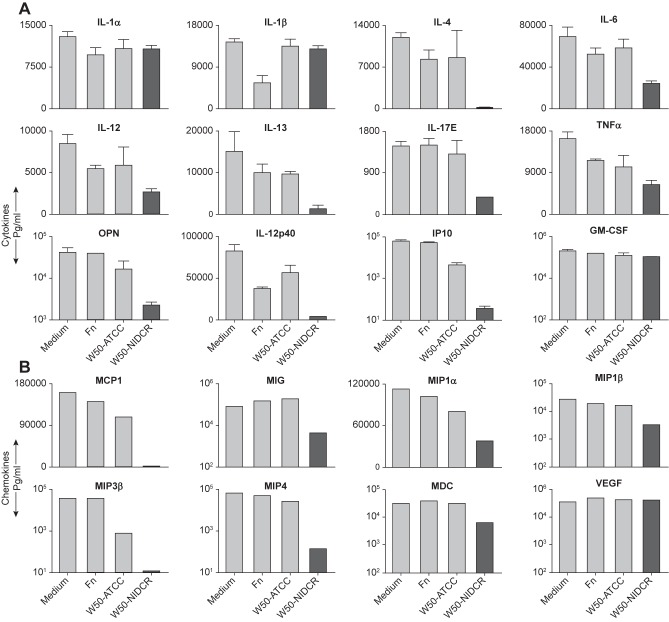
P. *gingivalis* supernatants also destroy human cytokines and chemokines. (**A**) Recombinant human cytokines were spiked and pooled into a single sample of 800 μl of complete medium (plus 10%FCS) in a 24-well plate in the absence (medium) or presence of 200 μl of particle-free bacterial SN and incubated at 37°C for 22 h, and the CNS were tested using human multiplex cytokine array. (**B**) Same as (A) except human chemokines were used. These data are expressed as the mean ± SD of representative of two independent experiments.

Since P. *gingivalis* is a human pathogen that does not normally infect mice, we asked if the destructive activity on mouse cytokines was an evolutionary useless feature or whether it would also hold for molecules involved in human immune responses. We tested a wide array of human cytokines and chemokines and found that all of them were sensitive to the action of the bacterial supernatant except IL-1 α, VEGF and GM-CSF (Fig 7). Collectively, these data demonstrate that inactivation of human cytokines are similar to mouse in which the supernatant from W50-NIDCR was more effective than that of W50-ATCC in inactivation of various human cytokines and chemokines.

## Discussion

The goal of this study was to examine the broad influence of oral bacteria on DC activation and cytokine production. In this study, we used two oral bacteria, P. *gingivalis* and F. *nucleatum*, and compared their properties to those of the well-studied bacterium E. *coli*. Using assays that directly measure DC functional responses, a series of genomic and proteomic analyses, a gingipain-specific enzyme assay and direct tests on mouse and human cytokines, we found that P. *gingivalis* dampens innate immune responses. First, P. *gingivalis* is not a strong activator of DCs. DCs cultured in the presence of P. *gingivalis* hardly upregulate costimulatory or antigen-presenting MHC molecules. They secreted only small amounts of inflammatory cytokines and did not contain high levels of mRNA for those cytokines in the presence of P. *gingivalis*. Second, P. *gingivalis* extinguishes DC activation induced by other bacteria. Because when DCs are activated by E. *coli* or F. *nucleatum* (a commensal oral bacterium) in the presence of IFNγ, they generally secrete copious amounts of inflammatory cytokines such as IL-12. However, if P. *gingivalis* was added to such cultures, the cytokine secretion induced by E. *coli* or F. *nucleatum* was suppressed. Because P. *gingivalis* secretes proteases such as the gingipains, that degrades many of the mouse and human cytokines that we had tested, this will ensure a non-response even if other local oral bacteria (such as F. *nucleatum*) induce immune responses. In fact, neighboring bacterial populations seem to benefit from the presence of P. *gingivalis*, as they tend to be increased in number (especially Staphylococcal, Streptococcal and Propionibacterial species), showing that the presence of P. *gingivalis* is not only a benefit to itself but also to other local bacteria [10].

Intriguingly, we found that P. *gingivalis*’s secreted products do not affect human IL-1 and GM-CSF (Fig 7A). In contrast, P. *gingivalis* had strong effects on mouse IL-1 β and GM-CSF (Fig 6). Whereas mouse IL-1 β contains an arginine (a target of gingipain) at position 126 (which would be exposed at the end of the first beta strand in the crystal structure of mouse IL-1 β(PDB id 8I1B), position 126 in the human IL-1 β is changed to threonine. Similarly, there are regions of mouse GM-CSF that contain arginine or lysine residues (also targets of gingipain) that have different amino acids in the respective position of the sequence in the human molecule; and these regions in the structure of mouse GM-CSF are on loops that can be easily targeted by the enzyme. There are several ways to interpret the resistance of the human molecules to the bacterial proteases. One possibility is that the human molecules may have evolved to resist degradation by gingipains as a mechanism to enhance inflammation to remove the bacterium. Another possibility is that the bacterium may have taken an advantage of changes in IL-1 to benefit its survival. Like many pathogenic bacteria, P. *gingivalis* require iron for its growth and virulence [47, 48], and this is accomplished by hydrolyzing hemoglobin [49, 50]. At high doses, IL-1 α causes capillary leakage [51, 52], which benefits the bacterium that relies on heme for its survival. Indeed, there are data suggesting that P. *gingivalis* induces high levels of IL-1 from human gingival epithelial cells [53]. Moreover, GM-CSF may also serve the bacterium, as it inhibits terminal differentiation of epithelial and endothelial cells and induces their growth [54, 55] thus, potentially providing a larger subgingival niche for the bacterium to survive. Combined, our data suggest that P. *gingivalis* evades/inactivate those molecules that will lead to its clearance and at the same time selectively spares those molecules that enhance its survival.

Finally, different studies on P. *gingivalis* have given different results. For example, it has been reported by Banchereau’s group, that the gums of people with gingivitis contained large numbers of immature/resting Langerhans cells [14], supporting our view that the bacterium is not an strong stimulator of DCs. In contrast, there are studies that have shown that very high doses of P. *gingivalis* LPS (doses that would have been lethal, had the LPS come from E coli), does stimulate DCs, but seems to prime T cells for the subsequent production of IL-5 rather than IFNγ [13, 14, 56]. In general, bacteria have more weapons that just the LPS. It has been shown that P. *gingivalis* (inside the cells) secretes a phosphatase (SerB) that prevents translocation of NF-kB to the nucleus (thus hindering cytokine secretion from gingival epithelial cells), several proteases that degrade complement components, and “capturing” ligands that bind complement antagonists [57]. Nevertheless, it has also been shown that in spite of the SerB-mediated suppression, gingival epithelial cells secrete inflammatory cytokines [58]. Our data suggest (perhaps not surprisingly) that the differences found from study to study might be due to the differences in the substrains of P. *gingivalis* that were used. The W50-NIDCR seems to be particularly effective at evading/dampening the secretion of innate and adaptive immune mediators.

Admittedly, this study has only begun to unravel what seems to be potentially deep nexus of interactions between W50 and DCs. While some of our observations suggest a linear correlation (eg. HagA and degradative ability of P. *gingivalis* supernatant), it is important to note that the biology of host-microbe interactions are complex. For instance, the composition of the P. *gingivalis* supernatant itself can be altered by vesiculation resulting from mutations in HagA secretory pathways. Indeed, as a reviewer has correctly pointed out, the mutation in the CTD region of HagA that we observed could also alter its localization. This can potentially short-circuit the ability of the PorSS pathway of CTD protein secretion, which will result in altered localization of gingipain proteinases and adhesins on the surface of bacterial cells. Future studies using more or less suppressive strain(s) isolated from patients may reveal which of these particular attributes correlate best with disease severity.

## Supporting information

S1 Fig DCs exposed to P. *gingivalis* are viable and capable of secreting IL-12 when stimulated with T cells. IL-12 can be elicited from resting or LPS-activated DCs by coculturing them with antigen-activated T cells [24]. To test whether DCs that have been cultured with P. *gingivalis* are viable or dead, we tested their ability to produce IL-12 when subsequently cocultured with activated 5C.C7 T cells [specific for Moth Cytochrome *c* (MCC)]. We also tested whether the presence of the bacteria in the DC/T-cell culture would inhibit secretion of IL-12. **(A**) DCs from B10.A-Rag2^-/-^ mice were pre-activated with F. *nucleatum*, P. *gingivalis* or nothing (medium) for 20 h, then washed and cocultured at 2x10^4^ cells/well with 5x10^5^ cell/well antigen-activated 5C.C7 T cells in the presence of 0.1 μM MCC peptide 83–103 in a 96-well plate at 37°C for 48 h. CSN were analyzed for the presence of IL-12 using specific ELISA. (**B)** Same as (A) except 5x10^7^ F. *nucleatum* or P. *gingivalis* were added to the coculture of DCs with T cells. These data are expressed as the mean ± SD of triplicate wells representative of two independent experiments.
(PDF)

S2 Fig Proteins identified in the SN from W50-ATCC by tandem mass spectrometry from Coomassie blue 1D gel. (PDF)
