# Supplementary figures and images for: Mechanisms by which Porphyromonas gingivalis evades innate immunity
Source: PLoS One. 2017 Aug 3;12(8):e0182164. doi: 10.1371/journal.pone.0182164 (PMC5542538; doi:10.1371/journal.pone.0182164)

Figure S1

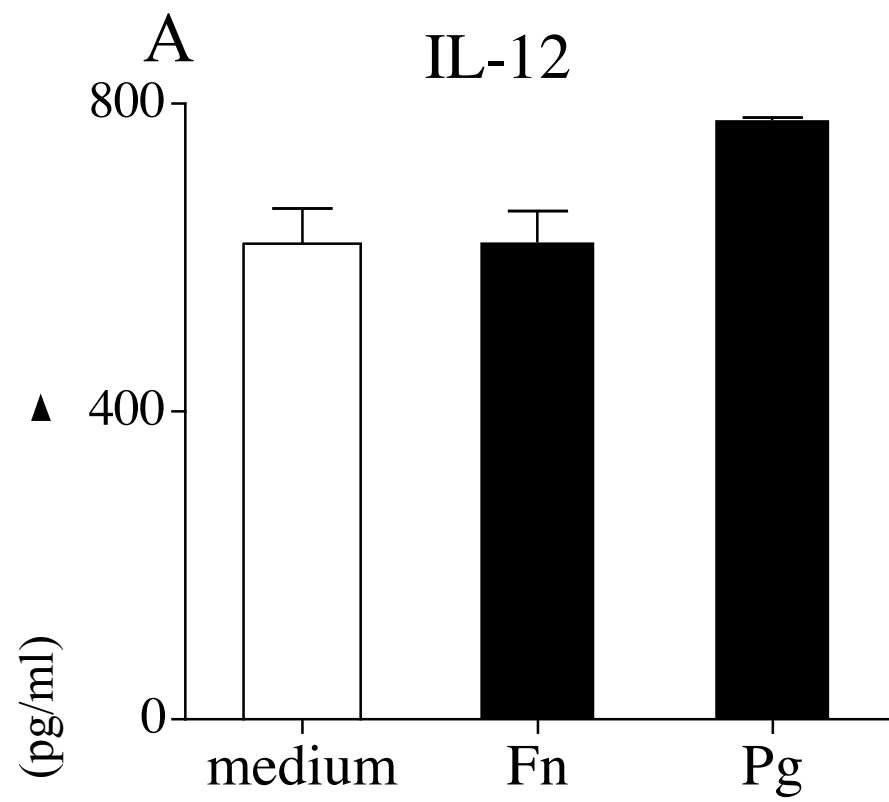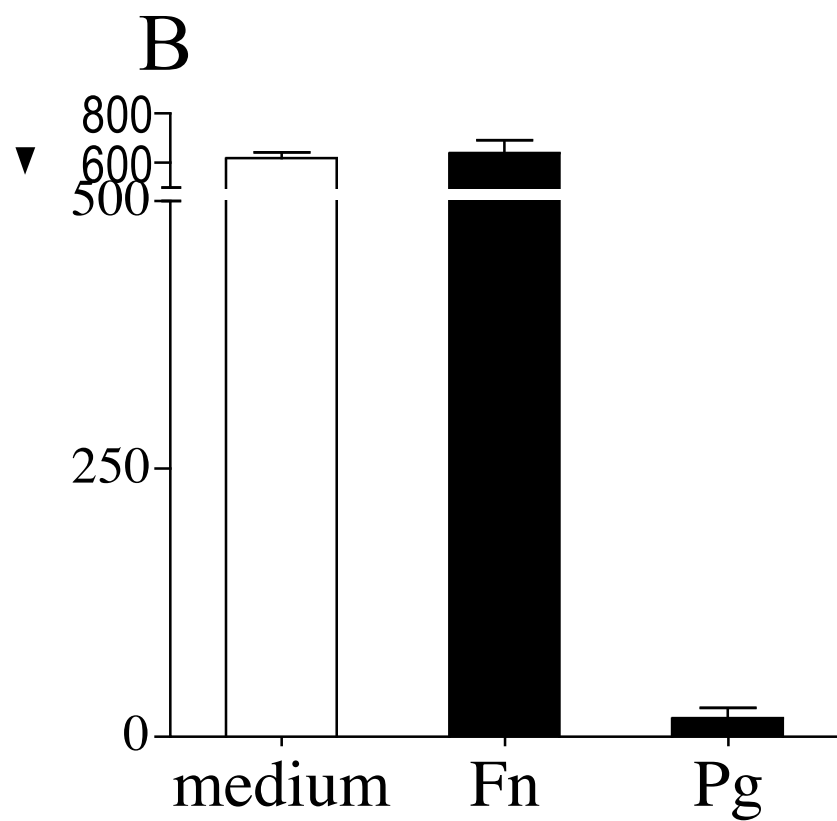

Supplement: S1 Fig — IL-12 can be elicited from resting or LPS-activated DCs by coculturing them with antigen-activated T cells [24]. To test whether DCs that have been cultured with P. gingivalis are viable or dead, we tested their ability to produce IL-12 when subsequently cocultured with activated 5C.C7 T cells [specific for Moth Cytochrome c (MCC)]. We also tested whether the presence of the bacteria in the DC/T-cell culture would inhibit secretion of IL-12. (A) DCs from B10.A-Rag2-/- mice were pre-activated with F. nucleatum, P. gingivalis or nothing (medium) for 20 h, then washed and cocultured at 2x10^4^ cells/well with 5x10^5^ cell/well antigen-activated 5C.C7 T cells in the presence of 0.1 μM MCC peptide 83–103 in a 96-well plate at 37°C for 48 h. CSN were analyzed for the presence of IL-12 using specific ELISA. (B) Same as (A) except 5x10^7^ F. nucleatum or P. gingivalis were added to the coculture of DCs with T cells. These data are expressed as the mean ± SD of triplicate wells representative of two independent experiments. (PDF) [file pone.0182164.s001.pdf]
