# Supplementary material for: Mechanisms by which Porphyromonas gingivalis evades innate immunity
Source: PLoS One. 2017 Aug 3;12(8):e0182164. doi: 10.1371/journal.pone.0182164 (PMC5542538; doi:10.1371/journal.pone.0182164)
Supplement: S2 Fig — (PDF) [file pone.0182164.s002.pdf]

Figure S2

BAND #1

	Reference					Score	Coverage	MW	Accession	Peptide (Hits)		% Height
	Scan(s)	Peptide	MH+	DeltaM	z	XC	DeltaCn	Sp	RSp	Ions	Count	Peak Height
1	receptor antigen (RagA) [Porphyromonas gingivalis]					460.33	42.40	112261.3	3901098	57 (57 0 0 0 0)		49.03
2	AF153768_1 immunoreactive 92 kDa antigen PG21 [Porphyromonas gingivalis]					250.31	28.00	91460.8	5059346	30 (30 0 0 0 0)		31.51
3	KGP83_PORGI RecName: Full=Lys-gingipain W83; AltName: Full=Lysine specific cysteine protease; AltName: Full=Lysine-specific cysteine protei					150.29	12.10	187756.8	75348574	16 (16 0 0 0 0)		1.42
4	PAD_PORGI RecName: Full=Peptidylarginine deiminase; Flags: Precursor					150.29	30.00	61690.5	73621345	17 (17 0 0 0 0)		6.45
5	AF175718_1 immunoreactive 32kD antigen PG49 [Porphyromonas gingivalis]					80.28	32.70	37089.1	5759285	8 (8 0 0 0 0)		0.70
6	AF237554_1 hypothetical tonB-linked outer membrane receptor PG50 [Porphyromonas gingivalis]					80.27	15.70	94396.0	8925305	8 (8 0 0 0 0)		0.77
7	PRTT_PORGI RecName: Full=Thiol protease/hemagglutinin prtT; Flags: Precursor					70.28	11.40	96386.1	1172653	7 (7 0 0 0 0)		0.84
8	AF200358_1 HmuY protein [Porphyromonas gingivalis]					40.29	52.10	15552.7	6409428	4 (4 0 0 0 0)		0.29
9	AF153770_1 immunoreactive 47 kDa antigen PG97 [Porphyromonas gingivalis]					40.18	11.00	47120.1	5059350	4 (4 0 0 0 0)		0.07
10	A Chain A, Crystal Structure Of The Arg Specific Cysteine Proteinase Gingipain R (Rgpb)					30.31	10.80	47853.8	7245522	4 (4 0 0 0 0)		0.88
11	CPG2_PORGI RecName: Full=Gingipain R2; AltName: Full=Arg-gingipain; AltName: Full=Gingipain 2; AltName: Full=RGP-2; Flags: Precursor					30.29	4.90	80916.2	37538303	4 (4 0 0 0 0)		0.24
12	RagA2 [Porphyromonas gingivalis]					30.26	3.20	115464.3	61652426	4 (4 0 0 0 0)		3.94
13	JC7920 35K hemin binding protein - Porphyromonas gingivalis					30.19	13.70	37536.4	60729604	3 (3 0 0 0 0)		0.20
14	RagA4 [Porphyromonas gingivalis]					20.24	2.20	114515.1	61652416	2 (2 0 0 0 0)		3.66

BAND #2

	Reference					Score	Coverage	MW	Accession	Peptide (Hits)		% Height
	Scan(s)	Peptide	MH+	DeltaM	z	XC	DeltaCn	Sp	RSp	Ions	Count	Peak Height
1	KGP83_PORGI RecName: Full=Lys-gingipain W83; AltName: Full=Lysine specific cysteine protease; AltName: Full=Lysine-specific cysteine protei					410.34	20.70	187756.8	75348574	66 (66 0 0 0 0)		57.07
2	unnamed protein product [Porphyromonas gingivalis]					270.40	63.70	56434.6	116636501	35 (35 0 0 0 0)		13.90
3	A Chain A, Crystal Structure Of The Arg Specific Cysteine Proteinase Gingipain R (Rgpb)					170.32	37.90	47853.8	7245522	20 (20 0 0 0 0)		7.77
4	JC7920 35K hemin binding protein - Porphyromonas gingivalis					130.28	34.30	37536.4	60729604	15 (15 0 0 0 0)		3.90
5	PAD_PORGI RecName: Full=Peptidylarginine deiminase; Flags: Precursor					120.27	26.30	61690.5	73621345	15 (15 0 0 0 0)		7.04
6	AF153770_1 immunoreactive 47 kDa antigen PG97 [Porphyromonas gingivalis]					110.20	29.70	47120.1	5059350	12 (12 0 0 0 0)		0.71
7	receptor antigen (RagA) [Porphyromonas gingivalis]					90.29	12.50	112261.3	3901098	9 (9 0 0 0 0)		0.37
8	KGP66_PORGI RecName: Full=Lys-gingipain HG66; Contains: RecName: Full=Lys-gingipain catalytic subunit; Contains: RecName: Full=39 kDa a					50.27	3.80	186713.3	75345025	6 (6 0 0 0 0)		3.03
9	AF200358_1 HmuY protein [Porphyromonas gingivalis]					50.26	52.80	15552.7	6409428	6 (6 0 0 0 0)		0.46
10	CPG2_PORGI RecName: Full=Gingipain R2; AltName: Full=Arg-gingipain; AltName: Full=Gingipain 2; AltName: Full=RGP-2; Flags: Precursor					40.30	6.40	80916.2	37538303	6 (6 0 0 0 0)		3.10
11	AF153768_1 immunoreactive 92 kDa antigen PG21 [Porphyromonas gingivalis]					40.23	5.70	91460.8	5059346	4 (4 0 0 0 0)		0.11
12	AF145801_1 immunoreactive 46 kDa antigen PG99 [Porphyromonas gingivalis]					30.26	12.30	45671.6	5081703	4 (4 0 0 0 0)		0.20
13	PRTT_PORGI RecName: Full=Thiol protease/hemagglutinin prtT; Flags: Precursor					30.22	4.30	96386.1	1172653	3 (3 0 0 0 0)		0.10
14	AF155223_1 tonB-linked receptor Tlr [Porphyromonas gingivalis]					30.21	6.20	78856.5	5007082	3 (3 0 0 0 0)		0.12
15	CPG1_PORGI RecName: Full=Gingipain R1; AltName: Full=Arg-gingipain; AltName: Full=Gingipain 1; AltName: Full=RGP-1; Flags: Precursor					20.31	4.60	108713.4	2827775	3 (3 0 0 0 0)		1.17
16	arginine-specific thiol protease precursor [Porphyromonas gingivalis]					20.25	1.20	185509.3	1066835	2 (2 0 0 0 0)		0.48
17	AF144641_1 immunoreactive 53 kD antigen PG123 [Porphyromonas gingivalis]					20.18	5.60	53491.0	4929297	2 (2 0 0 0 0)		0.06
18	B Chain B, Structure Of Porphyromonas Gingivalis Heme-Binding Protein Hmuy In Complex With Heme					20.17	11.00	21190.3	237640668	2 (2 0 0 0 0)		0.12
19	RagB4 [Porphyromonas gingivalis]					20.16	2.00	56866.6	61652417	2 (2 0 0 0 0)		0.29

BAND #3

	Reference					Score	Coverage	MW	Accession	Peptide (Hits)		% Height
	Scan(s)	Peptide	MH+	DeltaM	z	XC	DeltaCn	Sp	RSp	Ions	Count	Peak Height
1	AF200358_1 HmuY protein [Porphyromonas gingivalis]					210.37	83.10	15552.7	6409428	32 (32 0 0 0 0)		36.51
2	KGP83_PORGI RecName: Full=Lys-gingipain W83; AltName: Full=Lysine specific cysteine protease; AltName: Full=Lysine-specific cysteine protei					210.36	16.90	187756.8	75348574	25 (25 0 0 0 0)		5.82
3	AF145801_1 immunoreactive 46 kDa antigen PG99 [Porphyromonas gingivalis]					120.34	26.70	45671.6	5081703	17 (17 0 0 0 0)		2.86
4	A Chain A, Crystal Structure Of The Arg Specific Cysteine Proteinase Gingipain R (Rgpb)					110.31	25.50	47853.8	7245522	14 (14 0 0 0 0)		5.10
5	B Chain B, Structure Of Porphyromonas Gingivalis Heme-Binding Protein Hmuy In Complex With Heme					90.28	20.90	21190.3	237640668	13 (13 0 0 0 0)		37.79
6	unnamed protein product [Porphyromonas gingivalis]					90.27	24.60	56434.6	116636501	9 (9 0 0 0 0)		0.86
7	JC7920 35K hemin binding protein - Porphyromonas gingivalis					60.29	21.20	37536.4	60729604	7 (7 0 0 0 0)		0.60
8	AF155223_1 tonB-linked receptor Tlr [Porphyromonas gingivalis]					50.19	7.50	78856.5	5007082	5 (5 0 0 0 0)		0.46
9	KGP66_PORGI RecName: Full=Lys-gingipain HG66; Contains: RecName: Full=Lys-gingipain catalytic subunit; Contains: RecName: Full=39 kDa a					40.34	3.00	186713.3	75345025	5 (5 0 0 0 0)		1.16
10	CPG2_PORGI RecName: Full=Gingipain R2; AltName: Full=Arg-gingipain; AltName: Full=Gingipain 2; AltName: Full=RGP-2; Flags: Precursor					40.30	7.20	80916.2	37538303	5 (5 0 0 0 0)		2.08
11	receptor antigen (RagA) [Porphyromonas gingivalis]					40.24	4.20	112261.3	3901098	4 (4 0 0 0 0)		0.95
12	CPG1_PORGI RecName: Full=Gingipain R1; AltName: Full=Arg-gingipain; AltName: Full=Gingipain 1; AltName: Full=RGP-1; Flags: Precursor					30.28	4.60	108713.4	2827775	4 (4 0 0 0 0)		0.92
13	HAGA1_PORGI RecName: Full=Hemagglutinin A; Flags: Precursor					30.23	7.00	233241.6	38605638	3 (3 0 0 0 0)		0.29
14	arginine-specific thiol protease precursor [Porphyromonas gingivalis]					20.26	1.20	185509.3	1066835	2 (2 0 0 0 0)		0.28
15	hemin-binding protein 2 [Porphyromonas gingivalis]					20.26	7.40	23833.9	124053198	5 (5 0 0 0 0)		4.08
16	OMP40_PORGI RecName: Full=Outer membrane protein 40; Short=Omp40; AltName: Full=PG33; Flags: Precursor					20.23	8.20	42425.2	27805653	2 (2 0 0 0 0)		0.08
17	AF145799_1 immunogenic 23 kDa lipoprotein PG3 [Porphyromonas gingivalis]					20.20	11.70	22616.6	5081699	2 (2 0 0 0 0)		0.00
18	AF153770_1 immunoreactive 47 kDa antigen PG97 [Porphyromonas gingivalis]					20.17	4.70	47120.1	5059350	2 (2 0 0 0 0)		0.14

BAND #4

	Reference					Score	Coverage	MW	Accession	Peptide (Hits)		% Height
	Scan(s)	Peptide	MH+	DeltaM	z	XC	DeltaCn	Sp	RSp	Ions	Count	Peak Height
1	KGP83_PORGI RecName: Full=Lys-gingipain W83; AltName: Full=Lysine specific cysteine protease; AltName: Full=Lysine-specific cysteine protei					250.35	19.80	187756.8	75348574	31 (31 0 0 0 0)		18.23
2	A Chain A, Crystal Structure Of The Arg Specific Cysteine Proteinase Gingipain R (Rgpb)					130.32	28.30	47853.8	7245522	15 (15 0 0 0 0)		19.37
3	OMP41_PORGI RecName: Full=Outer membrane protein 41; Short=Omp41; AltName: Full=PG32; Flags: Precursor					80.26	20.50	43397.1	37538316	9 (9 0 0 0 0)		3.42
4	AF200358_1 HmuY protein [Porphyromonas gingivalis]					70.32	69.70	15552.7	6409428	9 (9 0 0 0 0)		3.75
5	unnamed protein product [Porphyromonas gingivalis]					70.25	16.20	56434.6	116636501	7 (7 0 0 0 0)		2.72
6	receptor antigen (RagA) [Porphyromonas gingivalis]					70.24	9.60	112261.3	3901098	7 (7 0 0 0 0)		3.63
7	casein alpha s1 [Bos taurus]					60.24	31.30	24513.4	30794348	8 (8 0 0 0 0)		8.69
8	CPG2_PORGI RecName: Full=Gingipain R2; AltName: Full=Arg-gingipain; AltName: Full=Gingipain 2; AltName: Full=RGP-2; Flags: Precursor					50.29	8.20	80916.2	37538303	6 (6 0 0 0 0)		3.45
9	HAGA1_PORGI RecName: Full=Hemagglutinin A; Flags: Precursor					50.23	7.80	233241.6	38605638	5 (5 0 0 0 0)		1.68
10	OMP40_PORGI RecName: Full=Outer membrane protein 40; Short=Omp40; AltName: Full=PG33; Flags: Precursor					50.23	15.80	42425.2	27805653	5 (5 0 0 0 0)		1.31
11	CPG1_PORGI RecName: Full=Gingipain R1; AltName: Full=Arg-gingipain; AltName: Full=Gingipain 1; AltName: Full=RGP-1; Flags: Precursor					30.27	4.60	108713.4	2827775	4 (4 0 0 0 0)		7.98
12	AF155223_1 tonB-linked receptor Tlr [Porphyromonas gingivalis]					30.27	6.20	78856.5	5007082	3 (3 0 0 0 0)		0.62
13	JC7920 35K hemin binding protein - Porphyromonas gingivalis					30.21	7.60	37536.4	60729604	3 (3 0 0 0 0)		0.48
14	keratin 71 [Homo sapiens]					30.18	6.10	57256.1	15618995	3 (3 0 0 0 0)		0.67
15	B Chain B, Structure Of Porphyromonas Gingivalis Heme-Binding Protein Hmuy In Complex With Heme					30.16	11.00	21190.3	237640668	3 (3 0 0 0 0)		1.33
16	KGP66_PORGI RecName: Full=Lys-gingipain HG66; Contains: RecName: Full=Lys-gingipain catalytic subunit; Contains: RecName: Full=39 kDa a					20.34	1.60	186713.3	75345025	3 (3 0 0 0 0)		22.50
17	RagB4 [Porphyromonas gingivalis]					20.11	2.00	56866.6	61652417	2 (2 0 0 0 0)		0.19

BAND #5

	Reference					Score	Coverage	MW	Accession	Peptide (Hits)		% Height
	Scan(s)	Peptide	MH+	DeltaM	z	XC	DeltaCn	Sp	RSp	Ions	Count	Peak Height
1	KGP83_PORGI RecName: Full=Lys-gingipain W83; AltName: Full=Lysine specific cysteine protease; AltName: Full=Lysine-specific cysteine protei					270.36	19.60	187756.8	75348574	38 (38 0 0 0 0)		69.48
2	A Chain A, Crystal Structure Of The Arg Specific Cysteine Proteinase Gingipain R (Rgpb)					110.31	21.80	47853.8	7245522	13 (13 0 0 0 0)		7.40
3	receptor antigen (RagA) [Porphyromonas gingivalis]					110.26	14.20	112261.3	3901098	12 (12 0 0 0 0)		2.64
4	DPS_PORGI RecName: Full=DNA protection during starvation protein					90.31	57.20	17853.3	229485357	9 (9 0 0 0 0)		2.64
5	AF200358_1 HmuY protein [Porphyromonas gingivalis]					50.31	45.10	15552.7	6409428	5 (5 0 0 0 0)		0.89
6	JC7920 35K hemin binding protein - Porphyromonas gingivalis					50.26	17.70	37536.4	60729604	5 (5 0 0 0 0)		0.87
7	unnamed protein product [Porphyromonas gingivalis]					50.21	40.60	18516.1	116636495	5 (5 0 0 0 0)		1.45
8	AF155223_1 tonB-linked receptor Tlr [Porphyromonas gingivalis]					40.22	7.80	78856.5	5007082	5 (5 0 0 0 0)		1.06
9	THIO_ECOLI RecName: Full=Thioredoxin-1; Short=Trx-1					30.20	39.40	11799.1	76363560	3 (3 0 0 0 0)		0.60

10	CPG1_PORGI RecName: Full=Gingipain R1; AltName: Full=Arg-gingipain; AltName: Full=Gingipain 1; AltName: Full=RGP-1; Flags: Precursor	30.20	5.20	108713.4	2827775	3 (3 0 0 0 0)		0.38
11	HAGA1_PORGI RecName: Full=Hemagglutinin A; Flags: Precursor	30.16	5.00	233241.6	38605638	3 (3 0 0 0 0)		1.02
12	KGP66_PORGI RecName: Full=Lys-gingipain HG66; Contains: RecName: Full=Lys-gingipain catalytic subunit; Contains: RecName: Full=39 kDa a	20.34	1.60	186713.3	75345025	3 (3 0 0 0 0)		1.22
13	PRTH_PORGI RecName: Full=Protease prtH	20.29	1.60	110168.0	1172651	3 (3 0 0 0 0)		6.85
14	CPG2_PORGI RecName: Full=Gingipain R2; AltName: Full=Arg-gingipain; AltName: Full=Gingipain 2; AltName: Full=RGP-2; Flags: Precursor	20.25	2.20	80916.2	37538303	3 (3 0 0 0 0)		2.73
15	OMP40_PORGI RecName: Full=Outer membrane protein 40; Short=Omp40; AltName: Full=PG33; Flags: Precursor	20.21	8.20	42425.2	27805653	2 (2 0 0 0 0)		0.30
16	RagA2 [Porphyromonas gingivalis]	20.19	2.30	115464.3	61652426	2 (2 0 0 0 0)		0.19
17	hemin-binding protein 2 [Porphyromonas gingivalis]	20.19	7.40	23833.9	124053198	2 (2 0 0 0 0)		0.07
18	unnamed protein product [Porphyromonas gingivalis]	20.15	6.40	56434.6	116636501	2 (2 0 0 0 0)		0.20

BAND #6

	Reference				Score	Coverage	MW	Accession	Peptide (Hits)		% Height
	Scan(s)	Peptide	MH+	DeltaM z	XC	DeltaCn	Sp	RSp	Ions	Count	Peak Height
1	KGP83_PORGI RecName: Full=Lys-gingipain W83; AltName: Full=Lysine specific cysteine protease; AltName: Full=Lysine-specific cysteine protei				230.37	18.20	187756.8	75348574	26 (26 0 0 0 0)		55.62
2	unnamed protein product [Porphyromonas gingivalis]				130.38	34.30	56434.6	116636501	14 (14 0 0 0 0)		3.26
3	AF155223_1 tonB-linked receptor Tlr [Porphyromonas gingivalis]				100.28	16.10	78856.5	5007082	10 (10 0 0 0 0)		0.83
4	JC7920 35K hemin binding protein - Porphyromonas gingivalis				100.25	25.60	37536.4	60729604	10 (10 0 0 0 0)		3.46
5	A Chain A, Crystal Structure Of The Arg Specific Cysteine Proteinase Gingipain R (Rgpb)				90.32	23.70	47853.8	7245522	11 (11 0 0 0 0)		7.57
6	CPG2_PORGI RecName: Full=Gingipain R2; AltName: Full=Arg-gingipain; AltName: Full=Gingipain 2; AltName: Full=RGP-2; Flags: Precursor				50.31	8.20	80916.2	37538303	7 (7 0 0 0 0)		7.16
7	AF144641_1 immunoreactive 53 kD antigen PG123 [Porphyromonas gingivalis]				50.22	13.40	53491.0	4929297	5 (5 0 0 0 0)		2.20
8	unnamed protein product [Porphyromonas gingivalis]				50.20	9.60	73112.1	300548609	5 (5 0 0 0 0)		1.52
9	AF200358_1 HmuY protein [Porphyromonas gingivalis]				40.27	36.60	15552.7	6409428	4 (4 0 0 0 0)		1.04
10	receptor antigen (RagA) [Porphyromonas gingivalis]				40.20	4.80	112261.3	3901098	4 (4 0 0 0 0)		0.18
11	PAD_PORGI RecName: Full=Peptidylarginine deiminase; Flags: Precursor				40.18	6.80	61690.5	73621345	5 (5 0 0 0 0)		0.76
12	AF153770_1 immunoreactive 47 kDa antigen PG97 [Porphyromonas gingivalis]				40.18	10.70	47120.1	5059350	4 (4 0 0 0 0)		0.93
13	PRTH_PORGI RecName: Full=Protease prtH				30.33	1.90	110168.0	1172651	5 (5 0 0 0 0)		9.51
14	THIO_ECOLI RecName: Full=Thioredoxin-1; Short=Trx-1				30.25	39.40	11799.1	76363560	3 (3 0 0 0 0)		0.66
15	arginine-specific thiol protease precursor [Porphyromonas gingivalis]				30.24	1.50	185509.3	1066835	3 (3 0 0 0 0)		0.97
16	CPG1_PORGI RecName: Full=Gingipain R1; AltName: Full=Arg-gingipain; AltName: Full=Gingipain 1; AltName: Full=RGP-1; Flags: Precursor				30.22	5.80	108713.4	2827775	4 (4 0 0 0 0)		1.17
17	KGP38_PORGI RecName: Full=Lys-gingipain 381; Contains: RecName: Full=Lys-gingipain catalytic subunit; Contains: RecName: Full=39 kDa adl				20.25	2.80	187143.7	75345024	2 (2 0 0 0 0)		1.50
18	SERC_PORGI RecName: Full=Phosphoserine aminotransferase; AltName: Full=Phosphohydroxythreonine aminotransferase; Short=PSAT				20.25	7.50	40148.6	73621876	2 (2 0 0 0 0)		0.35
19	AF145801_1 immunoreactive 46 kDa antigen PG99 [Porphyromonas gingivalis]				20.24	7.20	45671.6	5081703	3 (3 0 0 0 0)		0.32
20	AF237554_1 hypothetical tonB-linked outer membrane receptor PG50 [Porphyromonas gingivalis]				20.21	2.60	94396.0	8925305	2 (2 0 0 0 0)		0.12
21	unnamed protein product [Porphyromonas gingivalis]				20.16	16.90	18516.1	116636495	2 (2 0 0 0 0)		0.11
22	AF155351_1 immunoreactive 32 kD antigen PG25 [Porphyromonas gingivalis]				20.15	4.10	32252.1	5052029	2 (2 0 0 0 0)		0.76
